# *Klebsiella pneumoniae* detection by a light-controlled one-pot RPA-CRISPR/Cas12a method

**DOI:** 10.3389/fcimb.2025.1669860

**Published:** 2025-10-01

**Authors:** Lele Pan, Lijian Wei, Shihua Luo, Baoyan Ren, Miao Li, Lina Liang, Xuebin Li, Guijiang Wei

**Affiliations:** ^1^ Center for Medical Laboratory Science, Affiliated Hospital of Youjiang Medical University for Nationalities, Baise, Guangxi, China; ^2^ Key Laboratory of Research on Clinical Molecular Diagnosis for High Incidence Diseases in Western Guangxi of Guangxi Higher Education Institutions, Baise, Guangxi, China; ^3^ Baise Key Laboratory for Precise Genetic Testing of Long-dwelling Nationalities, Baise, Guangxi, China; ^4^ Engineering Research Center of Guangxi Higher Education Institutions for Precise Genetic Testing of Long-dwelling Nationalities, Baise, Guangxi, China; ^5^ Guangxi Engineering Research Center for Precise Genetic Testing of Long-dwelling Nationalities, Baise, Guangxi, China; ^6^ Department of Clinical Laboratory, Baise People’s Hospital, Affiliated Southwest Hospital of Youjiang Medical University for Nationalities, Baise, Guangxi, China; ^7^ Yaneng BlOscience (Shenzhen) Corporation, Shenzhen, Guangdong, China; ^8^ Modern Industrial College of Biomedicine and Great Health, Youjiang Medical University for Nationalities, Baise, Guangxi, China; ^9^ Clinical Genome Center, Guangxi KingMed Diagnostics, Nanning, Guangxi, China

**Keywords:** *Klebsiella pneumoniae*, recombinase polymerase amplification, one-pot, CRISPR/Cas12a, light-controlled

## Abstract

**Background:**

*Klebsiella pneumoniae* (*KP*) is a significant pathogenic bacterium responsible for severe infections in hospitals. However, existing traditional detection techniques, such as culture and PCR, are relatively inefficient. Therefore, this study aims to establish a rapid and convenient method for detecting *KP*.

**Methods:**

This study developed a single-tube detection method combining recombinant polymerase amplification (RPA) and light-controlled CRISPR/Cas12a. RPA primers were designed and screened for the *rcsA* gene of *KP* to effectively amplify the target. A light-controlled CRISPR/Cas12a system was created using crRNA modified with a photocleavable group (NPOM). The two systems were integrated into a single tube. Following RPA amplification, UV light-controlled release of crRNA inhibition activates CRISPR-mediated target recognition and Cas12a trans-cleavage, detecting fluorescent signals (FD) in conjunction with UV analysis.

**Results:**

The light-controlled RPA-CRISPR/Cas12a detection platform developed in this study uses a 15 μL reaction system. By optimizing key parameters such as RPA amplification time (20 min), primer concentration (400 nM), UV light activation time (30 s), and crRNA/Cas12a concentration (300 nM), the platform achieves optimal detection efficiency. The platform has a fluorescence detection limit of 4.072×10^2^ copies/reaction and can specifically identify *KP* in seven common clinical strains. Clinical sample validation demonstrated that the method yields results fully consistent with PCR detection (30/30 agreement rate of 100%), showcasing excellent detection performance and clinical application potential.

**Conclusion:**

We have successfully developed a light-controlled RPA-CRISPR/Cas12a detection system capable of rapidly and highly sensitively detecting *KP*. This system demonstrates significant advantages in terms of detection speed (completed in as little as 50 minutes), sensitivity (as low as 4.072×10^2^ copies/reaction), and ease of use, providing an efficient and reliable solution for clinical pathogen detection.

## 1 Introduction

*KP*, a Gram-negative bacterium that belongs to the Enterobacteriaceae family ubiquitously found on mucosal surfaces of animals and in the environment (e.g., water, soil, etc.) (Adeolu et al., 2016; Paczosa and Mecsas, 2016). In humans, the bacterium mainly colonizes the gastrointestinal tract, and a few can be detected in the nasopharynx. As a commensal and opportunistic pathogen, *KP* is an important causative agent of community-acquired infections and can also trigger severe hospital-acquired infections. In immunocompromised patients, the bacterium can lead to a variety of serious diseases such as urinary tract infections, stroke-associated pneumonia, bacteremia, liver abscesses, and sepsis (Chen et al., 2013; Yao et al., 2015). Clinical surveillance data in China showed that *KP* accounted for 11.9% of pathogens from intensive care unit-acquired pneumonia and ventilator-associated pneumonia (Zhang et al., 2014). In addition, a multicenter study covering 25 hospitals in 14 provinces in China showed that *KP* was detected in up to 73.9% of 664 clinical samples of carbapenem-resistant Enterobacteriaceae (Zhang et al., 2018). This organism is also one of the main causative pathogens of neonatal sepsis, ranking among the top three in most case statistics (Okomo et al., 2019). Its high morbidity and lethality impose a heavy burden on our public health. Of concern is the spread of carbapenem-resistant *KP* in recent years, which has exacerbated the difficulty of clinical treatment (Zhang et al., 2015). Therefore, the timely identification of *KP* is particularly crucial.

The currently available detection approaches for *KP* exhibit several inherent constraints. Traditional bacterial culture methods, although reliable as the gold standard, demand no less than 24 hours of incubation time (Khan et al., 2014). Matrix-assisted laser desorption ionization time-of-flight mass spectrometry provides superior identification accuracy but still relies on the incubation step (Huang et al., 2022). Among the molecular detection techniques, polymerase chain reaction (PCR) reduces detection time, but reliance on expensive instruments and specialized personnel limits its application at the grassroots level (Dai et al., 2019).

In recent years, isothermal nucleic acid amplification technologies (for example, loop-mediated isothermal amplification (LAMP), recombinase polymerase amplification (RPA), and rolling circle amplification (RCA), etc.) have radically eliminated the reliance on complex thermal cycling equipment by utilizing a specific enzyme/primer system to complete the amplification at constant temperatures, dramatically simplifying the operation process and significantly reducing the reliance on specialized instruments and environments, which greatly expands the applicability and accessibility of the nucleic acids for rapid detection (Dong et al., 2015; Si et al., 2021). Compared to LAMP and RCA technologies, RPA offers advantages of simpler primer design and higher amplification efficiency. Specifically, LAMP requires multiple primer pairs and has a longer reaction time; RCA relies on circular templates and can only perform linear amplification, resulting in lower product yields (Srivastava and Prasad, 2023).

CRISPR/Cas system molecular diagnostic technology has become an emerging detection tool due to its high sensitivity, specificity, and convenience. The CRISPR/Cas system can specifically recognize the target nucleic acid sequences by guide RNAs (crRNAs) and activate collateral cleavage activity of Cas proteins, including Cas12 and Cas13. Collateral cleavage activity non-specifically degrades fluorescent reporter molecules and generates detection signals (Gootenberg et al., 2017; Chen et al., 2018). The RPA-CRISPR/Cas technology integrates the rapidity of isothermal amplification with the high specificity of CRISPR/Cas. Based on this advantage, researchers have developed various RPA-CRISPR/Cas diagnostic platforms, such as DNA Nucleic Acid Endonuclease Targeted CRISPR Reporter System (DETECTR), High Sensitivity Enzymatic Unlocking Reporter System (SHERLOCK), and High Efficiency and Low Consumption Multi-Purpose System (HOLMES), which are technologies with single-base resolution and high sensitivity at the molar level (Li et al., 2018b; Kellner et al., 2019; Lin et al., 2023; Lan et al., 2024; Sen et al., 2024; Xue et al., 2025). Therefore, “one-pot” assays that combine the two steps of traditional RPA-CRISPR/Cas technology into a single reaction tube are particularly important (Harrington et al., 2018; Li et al., 2018a).

Li et al. leveraged plant suction dynamics to isolate and link the RPA-CRISPR/Cas reaction mixture in the channel, enabling a one-pot assay; however, the technique is laborious and contamination-prone (Chen et al., 2025). Liu and Hu et al. proposed an improved approach by loading amplification reagents into the bottom of reaction tubes and fixing Cas12a/crRNA complexes on the tube caps to form a compartmentalized system, effectively avoiding premixing (Hu J.J, et al., 2023). Nevertheless, sensitivity still has room for improvement. Subsequently, Zhang, Tan, and Wang teams introduced high-viscosity additives (e.g., glycerol, paraffin, sucrose) to achieve spatial separation, enhancing simplicity and efficiency (Tan Q. et al., 2024; Wang et al., 2024; Zhang et al., 2024). However, the reproducibility of this method may be compromised by the disruptive effects of high-viscosity additives on the solution phase. Building on significant advances in “one-pot” nucleic acid detection technology, the Hu and Chen team. Employed light-cleavable linkers to block crRNA, achieving spatiotemporal separation in a single-tube closed system (Chen et al., 2022; Hu et al., 2022). However, the additional hybridization step limited the development of lyophilized reagents. To address this, Hu et al. Further optimized the approach by incorporating the photocleavable base NPOM-dt into crRNA, eliminating the pre-hybridization step and providing a feasible solution for future lyophilized reagent development (Hu et al., 2023). To date, the detection of *KP* using a light-controlled RPA-CRISPR/Cas12a using NPOM-dt has not yet been reported.

In this study, we plan to design RPA primers based on the conserved gene *rcsA* of *KP* and develop a single-tube rapid detection system based on light-controlled RPA-CRISPR/Cas12a using NPOM-dt. The method adopts a tube-closed reaction without the addition of extra reagents, and triggers the CRISPR/Cas12a system by ultraviolet light irradiation after RPA amplification, achieving simple, rapid, and highly specific detection of *KP*.

## 2 Materials and methods

### 2.1 Materials

The standard strains used in this study are as follows: *KP* (ATCC 700603), *Escherichia coli* (ATCC 25922), *Pseudomonas aeruginosa* (ATCC 27853), *Streptococcus pneumoniae* (ATCC 49619), *Enterococcus faecalis* (ATCC 29212), and *Staphylococcus aureus* (ATCC 29213). The above strains were provided by the Laboratory Strain Bank. *Enterococcus faecalis* (GDMCC 1.388) was procured from Guangdong Microbiology, and clinical strains of *Haemophilus parainfluenzae* were isolated and confirmed by the Microbiology Unit of the Department of Laboratory Medicine. Genomic DNA extraction for all strains was performed in a molecular biology laboratory. Details of strain sources are shown in **Supplementary Table S1**. The standard RPA kit (TwistDx Company Limited, UK) was used for nucleic acid amplification. Cas12a (Cpf1) protein (EnGen^®^ Lba) and the corresponding NEBuffer r2.1 were procured from New England Biolabs (China branch). PCR reactions were performed using 2X Taq MasterMix (with dye) from Cwbio (Jiangsu, China). The TIANamp Bacterial DNA Kit (Tiangen, China) was employed for bacterial genomic DNA extraction. **Supplementary Table S2** summarizes the equipment specifications for fluorescence recording, gel documentation, and UV-based detection. Most oligonucleotides in this study, including PCR primers, cRNA, RPA primers, and ssDNA (single-stranded DNA), were purchased and synthesized from Nanning GenSys Biotechnology Co. Caged crRNA was purchased from Bolles Biosciences (Guangzhou, China). Ultraviolet lamp (365 nm) was purchased from Zhonglian UV Optics Factory (Shenzhen, China).

### 2.2 Genomic DNA extraction

For long-term preservation, the reference strains were stored at -80°C in 20% glycerol. Before the experiment, the reference strain was streaked onto blood agar plates and incubated at 37 °C overnight (approximately 16–24 h). A single colony was placed in a 1.5 mL EP tube, and genomic DNA was extracted using the TIANamp Bacterial DNA Extraction Kit. Clinical strains were preserved in skimmed milk medium at -80 °C, and DNA extraction was also done using the TIANamp kit. The concentration of all extracted DNA samples was determined spectrophotometrically, and the OD260/280 ratio of eligible samples was between 1.8 and 2.0. Bacterial genomic DNA that was not used immediately was stored at -80 °C for backup, whereas DNA templates before the experiment were temporarily stored at -20 °C for use.

### 2.3 Primer, crRNA, and caged crRNA design

Based on the *rcsA* gene sequence of the KP strain obtained from the NCBI database (GenBank: AY059955.1), three pairs of RPA primers (*rcsA* Pair1, *rcsA* Pair3, and *rcsA* Pair4) using the NCBI online “Primer-BLAST” tool. The primer pairs designed by previous researchers (Tan et al., 2024a) were selected as *rcsA* Pair2 for screening. The primer design parameters were set as follows: length 28–35 bp, GC content 30-70%, amplicon length 150–300 bp, Tm value 50-100%, and maximum single-nucleotide repeat length not more than 5. After screening the primers and verifying specificity via 2% agarose gel electrophoresis, we determined the optimal RPA primer combinations. Subsequently, three sets of crRNAs were designed by identifying PAM (protospacer adjacent motif, TTTN) sites in the amplification region of the selected primers using CRISPR online tools (http://www.rgenome.net/cas-designer), and the most effective crRNAs were screened by Cas12a-mediated fluorescence signal detection. Finally, the photocleavable group 6-nitropiperonyloxymethyl-caged thymidine (NPOM-dt) was introduced into the spacer region of the selected optimal crRNA sequence, resulting in the construction of an activity-modulable caged crRNA (Hu et al., 2023). As a photocleavable group, NPOM-dt modification temporarily inactivates caged crRNA, inhibiting Cas12a activity. Upon UV irradiation of caged crRNA, Cas12a cleavage function will be activated. The details of primers, crRNA, ssDNA, and caged crRNA used in this work are provided in **Supplementary Table S3**.

### 2.4 RPA primer optimization and specificity validation

RPA reactions were performed in strict accordance with the Twist AmpTM Basic Kit instructions, and contained 2.4 μL (10 μM) of forward and reverse primers, 29.5 μL of buffer, 4 μL of DNA template, 2.5 μL of magnesium ions (incorporated at the end to avoid non-specific amplification), and lyophilized enzyme powders (polymerase, single-stranded binding protein, and recombinase, etc.), and the volume was made up using enzyme-free water. To avoid loss of enzyme activity, lyophilized enzyme powders should be centrifuged briefly before use, and repeated freezing and thawing should be avoided. The reaction tubes were mixed, centrifuged, and incubated in a PCR machine at 37 °C for 20 min. Amplification products were then resolved on a 2% agarose gel (130 V, 45 min) for analysis. Primer specificity verification was tested by using the same reaction system and only replacing different DNA templates.

### 2.5 Establishment of a light-controlled one-pot RPA-CRISPR/Cas12a method

Firstly, to screen the optimal crRNA, a Cas12a-mediated fluorescence detection assay was performed. The reaction system (20 μL) incorporated 2 μL of 10×NEBuffer, 500 nM FAM-BHQ1-tagged reporter molecule (5’-FAM-TTATT-BHQ1-3’), 50 nM Cas12a protein, 50 nM crRNA, and enzyme-free water, and 2 μL of RPA amplification product was added. 2 μL of RPA amplification product was followed by monitoring the fluorescence signal for 30 min at 37 °C (one acquisition per minute), and the data were analyzed by GraphPad Prism 9 to determine the optimal crRNA. Three NPOM-dt modifications were subsequently embedded in its spacer region to generate caged crRNA. Based on this, we designed the light-controlled single-tube RPA-CRISPR/Cas12a system to detect *KP*. The reaction system consisted of a 15 μL mixture (containing 10 μL of RPA solution: 5.9 μL of 2× buffer, 0.48 μL of forward/reverse primer (10 μM), 0.5 μL of magnesium acetate, and enzyme-free water to make up to 10 μL) and 5 μL of light-controlled CRISPR/Cas12a components (1 μL of 10× buffer, 1 μL of Cas12a protein (4 μM), 1 μL of caged crRNA (4 μM), 1 μL FAM-BHQ1 probe (10 μM) and enzyme-free water made up to 5 μL) were composed. After mixing and centrifugation, the reaction tubes were incubated at 37 °C for 20 min, and the crRNA was activated by ultraviolet light irradiation for 30 s. The fluorescence assay was then continued at 37 °C for 30 min (recorded every minute). The feasibility of the method was verified by deletion experiments (ultraviolet light irradiation, template, Cas12a, caged crRNA, or probe).

### 2.6 Optimization of the light-controlled one-pot RPA-CRISPR/Cas12a system

In order to achieve optimal amplification efficiency of the method, systematic optimization of reaction parameters was essential. First, the amplification conditions were systematically evaluated, including testing different amplification times (10 min, 15 min, 20 min, 30 min, 40 min) and primer concentration gradients (160 nm, 240 nm, 320 nm, 400 nm, 480 nm, 560 nm) to determine the optimal amplification efficiency. Meanwhile, the ultraviolet light irradiation time (10s, 15s, 20s, 30s, 60s) was finely regulated to ensure adequate activation of caged crRNA. In addition, the concentrations of Cas12a (100–500 nM) and caged crRNA (100–400 nM) were optimized under different concentration conditions to maximize the target cleavage efficiency and minimize non-specific cleavage by maintaining the initial reaction volume constant. All optimization experiments were performed with fluorescence intensity as the key assessment metric, a blank reaction was set up as a negative control, and real-time fluorescence monitoring was performed by a qPCR instrument, and each parameter combination was repeated three times to ensure data reliability.

### 2.7 Sensitivity and specificity evaluation of the light-controlled one-pot RPA-CRISPR/Cas12a method

The sensitivity and specificity of the light-controlled “one-pot” RPA-CRISPR/Cas12a system were evaluated. A 10-fold gradient dilution (4.072×10^6^ copies/reaction to 4.072×10^2^ copies/reaction) of *KP* genomic DNA was used as a template for sensitivity testing using ddH2O as diluent. Subsequently, system specificity was examined using standardized genomic DNA of *KP* and seven other common clinical pathogens, including *Haemophilus parainfluenzae*, *Pseudomonas aeruginosa*, *Streptococcus pneumoniae*, *Enterococcus faecalis*, *Enterococcus aureus*, and *Escherichia coli*. All experiments were repeated three times.

### 2.8 Clinical evaluation of the light-controlled one-pot RPA-CRISPR/Cas12a method

To evaluate diagnostic performance, 30 *KP* and 20 *non-KP* samples were obtained from clinical laboratories. After obtaining genomic DNA using a commercial DNA extraction kit, the light-controlled single-tube RPA-CRISPR/Cas12a system was simultaneously validated against conventional PCR techniques, with statistical analysis performed using GraphPad Prism software(Version 10.4, La Jolla, California, USA). The total volume of the PCR reaction system was 25 μL, which consisted of 12.5 μL of 2× Es Taq MasterMix (Dye), 0.4 μM upstream and downstream primers, and 2 μL of DNA template. The PCR amplification program was set up strictly according to the CWBIO 2×Es Tap MasterMix (Dye) reagent instructions. The amplification products of the two methods were finally analyzed by 2% agarose gel electrophoresis. All experiments were repeated three times.

## 3 Result

### 3.1 The workflow for the detection of KP by light-controlled one-pot RPA-CRISPR/Cas12a method

**Figure 1:**
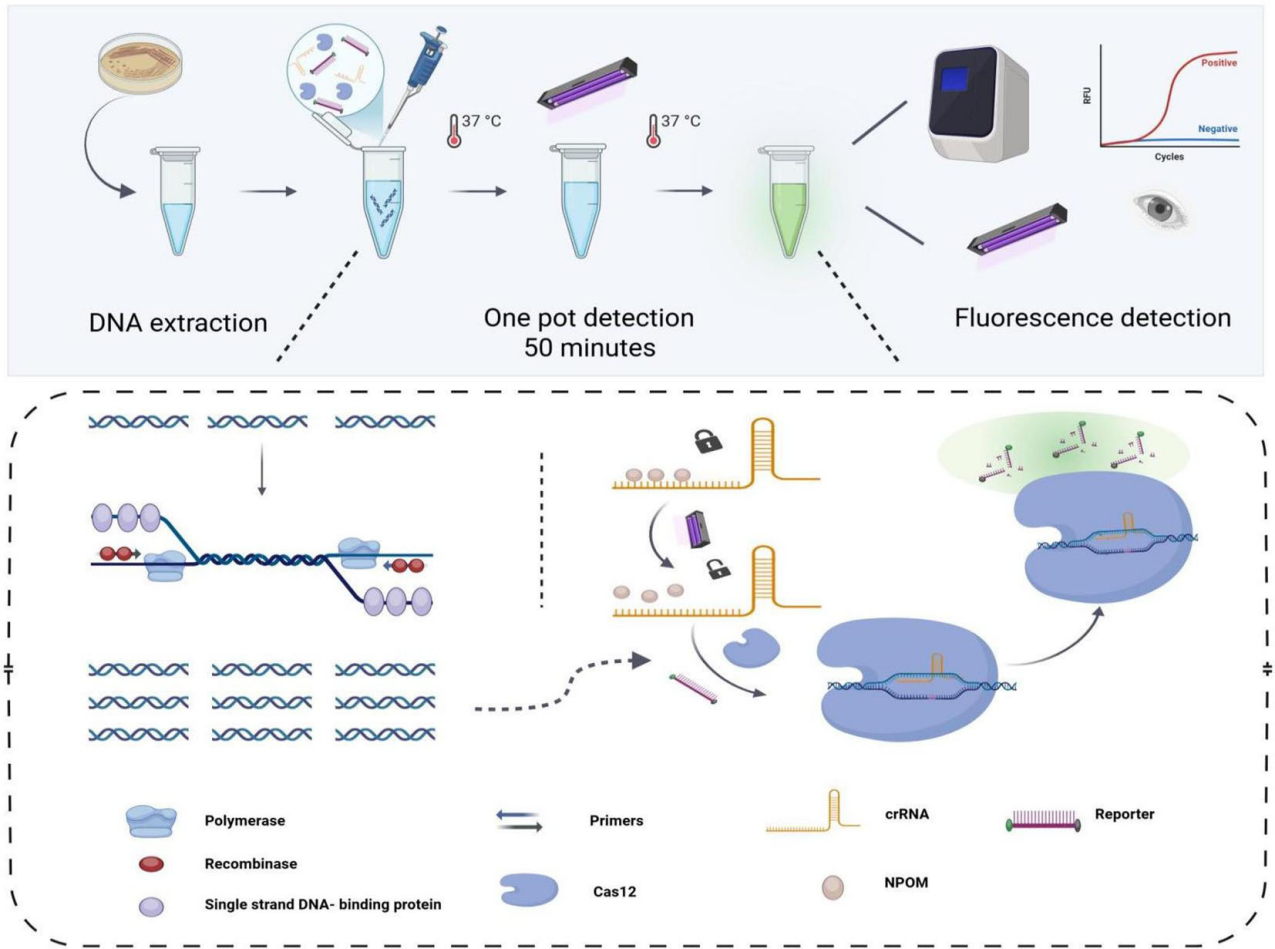
The workflow of the one-tube light-controlled RPA-CRISPR/Cas12a method. The template is introduced into a reaction tube containing both the RPA amplification system and the light-controlled CRISPR/Cas12a detection system. Subsequently, the CRISPR/Cas12a detection system is activated by ultraviolet light irradiation to perform specific and nonspecific cleavage of the template and the reporter DNA, respectively. Ultimately, the detection outcomes are interpretable using either a fluorescence detector or an ultraviolet (UV) detection system.

As illustrated in **Figure 1**, the workflow for the detection of *KP* by the light-controlled “one-pot” RPA-CRISPR/Cas12a method. First, a 20-minute isothermal amplification of RPA was performed without ultraviolet light irradiation, which was performed by a synergistic combination of recombinase, single-stranded binding proteins, and specific primers. After the target fragment was fully amplified, it was precisely irradiated by 360 nm ultraviolet light on the tube wall for 30 seconds to promote the efficient dissociation of the NPOM-dt protective group on the crRNA and realize the functional activation of crRNA. The activated crRNA-Cas12a complex first recognizes the PAM sequence of the target DNA and then binds specifically by complementary base pairing, triggering the double cleavage activity of Cas12a, which can both precisely cleave the DNA strand of interest and non-specifically cleave the ssDNA fluorescent reporter molecule in the system. The detection results are directly interpreted by real-time fluorescence signals, and the design realizes the spatiotemporal isolation of amplification and detection, providing a non-contact, one-step solution for the point-of-care detection of nucleic acids.

### 3.2 RPA primer screening and specificity validation

**Figure 2:**
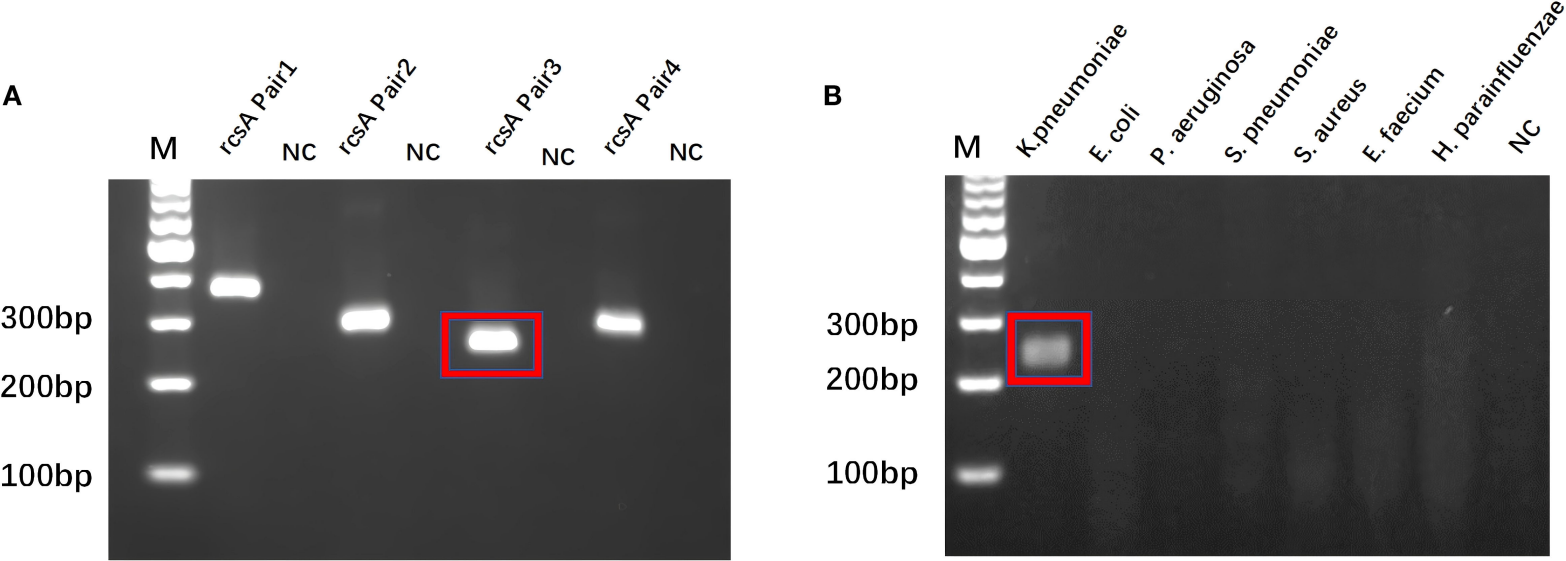
RPA primer screening and specificity validation. **(A)** Best RPA Primer Screening: *rcsA* Pair 3 is the best RPA primer. **(B)** Specificity verification of *rcsA* Pair 3. M, Marker; NC, Negative Control.

The *rcsA* gene is a specific gene of *KP* (Tan et al., 2024a). Based on the *rcsA* gene sequence, three pairs of RPA primers were designed in our research (**Supplementary Table S3**). Amplification reactions and electrophoresis analysis verified that each primer was effective in identifying the target sequence (**Figure 2A**). From the imaging results, it can be observed that *rcsA* Pair3 has the highest amplification product yield, and the specificity verification is satisfactory. The cross-reactivity experiment confirmed (**Figure 2B**) that the primer pair was only amplified to the target template, so the Pair3 amplification sequence was identified as critical for designing crRNA and downstream experiments.

### 3.3 Establishment and performance evaluation of light control one-pot RPA-CRISPR/Cas12a method

**Figure 3:**
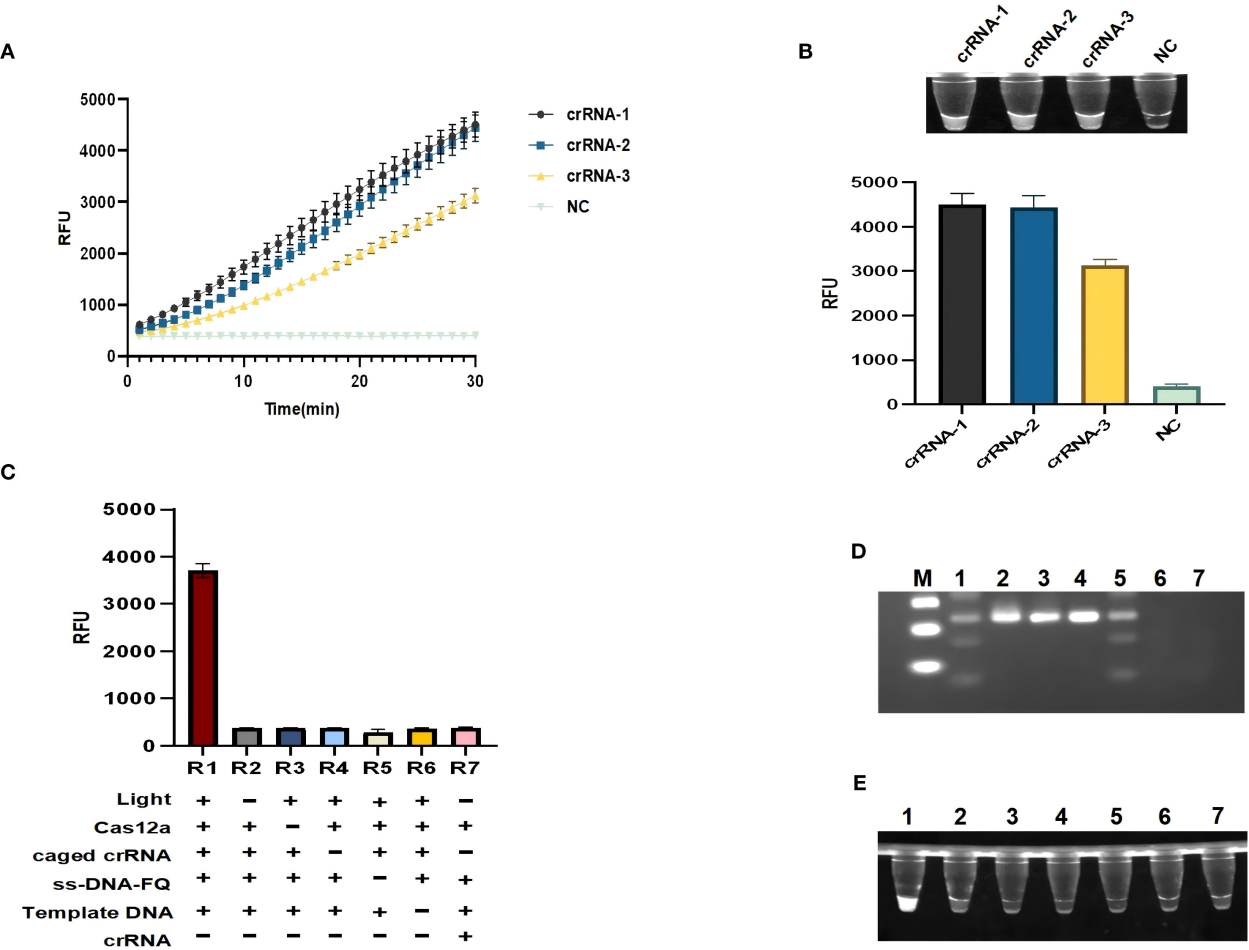
Establishment of a light-controlled one-pot RPA-CRISPR/Cas12a method. **(A, B)** Fluorescence curves and endpoint signals of three crRNAs designed for the *rcsA* amplicon. N = 3, error bars represent standard deviation. **(C)** Component omission experiments to validate the feasibility of the light-controlled one-pot RPA-CRISPR/Cas12a method.(+) represents the added reaction component, (-) represents the not added reaction component.R1:Light (+), Cas12a(+), caged crRNA(+), ss-DNA-FQ(+), Template DNA(+), crRNA (-); R2:Light (-), Cas12a(+), caged crRNA(+), ss-DNA-FQ(+), Template DNA(+), crRNA (-); R3:Light (-), Cas12a (-), caged crRNA(+), ss-DNA-FQ(+), Template DNA(+), crRNA (-); R4:Light (-), Cas12a(+), caged crRNA(-), ss-DNA-FQ(+), Template DNA(+), crRNA (-); R5:Light (-), Cas12a(+), caged crRNA(+), ss-DNA-FQ(-), Template DNA(+), crRNA (-); R6:Light (-), Cas12a(+), caged crRNA(+), ss-DNA-FQ(+), Template DNA(-), crRNA (-); R7:Light (-), Cas12a(+), caged crRNA (-), ss-DNA-FQ(+), Template DNA(-), crRNA (+). **(D)** Gel electrophoresis analysis under the same corresponding conditions as **(C)**. **(E)** Ultraviolet imaging showing the nonspecific cleavage of the light-controlled one-pot RPA-CRISPR/Cas12a method. M, marker; NC, negative control.

Three crRNAs were designed based on the amplified fragments, and each crRNA successfully activated the specific recognition of Cas12a protein, but only *rcsA*-crRNA1 (**Figures 3A, B**) showed the highest non-specific cleavage efficiency at 30 min for designing caged crRNAs for subsequent experiments. We systematically excluded reaction components to assess the robustness of the light-regulated “one-pot” RPA-CRISPR/Cas12a assay. As shown in **Figure 3C**, the observed fluorescence signal is negligible in the absence of Cas12a, probes, target DNA, illumination, or caged crRNA, including the traditional “one-pot” method. However, only when all reaction components are present simultaneously and irradiated with ultraviolet light, the fluorescence signal intensity significantly increases, and specific and non-specific cleavage (trans-cleavage) functions are activated (**Figures 3D, E** **).**

### 3.4 Optimization of the light-controlled one-pot RPA-CRISPR/Cas12a method

**Figure 4:**
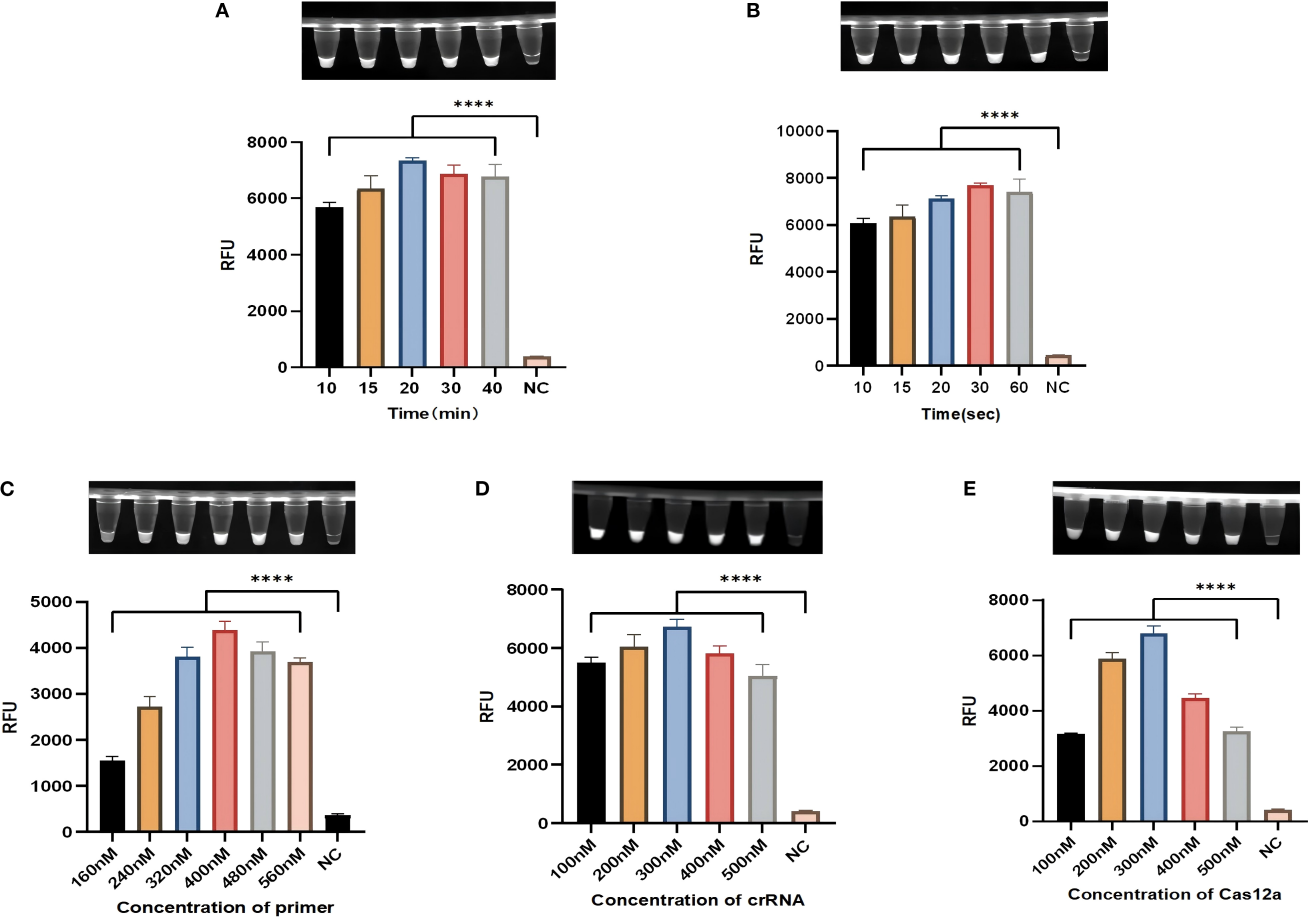
Optimization of the light-controlled one-pot RPA-CRISPR/Cas12a method. **(A, C)** The amplification time and primer concentration in the light-controlled one-pot RPA-CRISPR/Cas12a system. **(B)** The irradiation time in the light-controlled one-pot RPA-CRISPR/Cas12a system. **(D, E)** The caged crRNA concentration and Cas protein concentration in the light-controlled one-pot RPA-CRISPR/Cas12a system. N = 3, error bars represent the standard deviation. NC, negative control. Two-tailed Student’s t-test, ****p < 0.0001.

To improve the sensitivity of the diagnostic method, the conditions were optimized. Firstly, the RPA amplification system was optimized. Optimization focused on testing different amplification times (10, 15, 20, 30 and 40 min) and primer concentrations (160, 240, 320, 400, 480 and 560 nM). When the primer concentration is 400 nM and amplified for 20 min, the amplification efficiency is the best, and the fluorescence efficiency is the highest (**Figures 4A, C**). The light-controlled Cas12a detection system was then optimized, and the fluorescence values showed that the fluorescence efficiency was best at 300 nM for the caged crRNA and 300 nM for the Cas protein (**Figures 4D, E**). In order to fully activate the caged crRNA, we optimized the light time. At 30 s of illumination, the fluorescence values were highest (**Figure 4B**).

### 3.5 Sensitivity and specificity of the light-controlled one-pot RPA-CRISPR/Cas12a method

**Figure 5:**
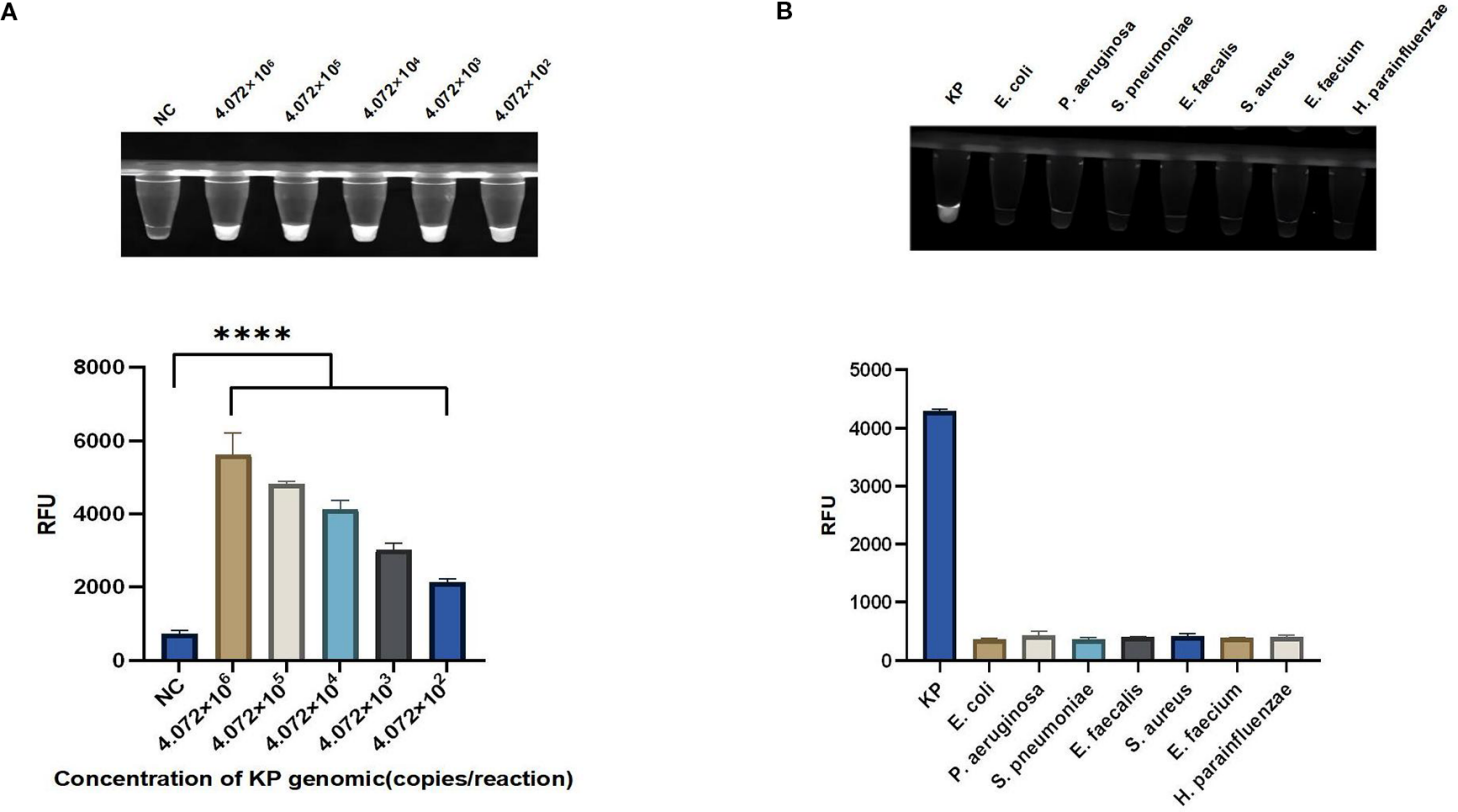
Sensitivity and specificity of the light-controlled one-pot RPA-CRISPR/Cas12a method. **(A)** Serial dilutions of the *KP* genomic DNA were used to validate the sensitivity of the light-controlled one-pot RPA-CRISPR/Cas12a system. **(B)** The specificity of the light-controlled one-pot RPA-CRISPR/Cas12a system was validated using seven common clinical pathogens. N = 3, error bars represent the standard deviation. NC, negative control. Two-tailed Student’s t-test, ****p < 0.0001.

The sensitivity of the light-controlled one-tube RPA-CRISPR/Cas12a system was evaluated by serial dilutions of *KP* genomic DNA from 4.072×10^6^ copies/reaction to 4.072×10^2^ copies/reaction. The above research results indicate that the single-tube RPA-CRISPR/Cas12a system under light control may have higher sensitivity in detecting *KP* (**Figure 5A**). To determine the specificity of the light-controlled one-tube RPA-CRISPR/Cas12a system, we tested for common clinical pathogens, including *KP*, *Haemophilus parainfluenzae*, *Pseudomonas aeruginosa*, *Streptococcus pneumoniae*, *Enterococcus faecium*, *Enterococcus faecalis*, *Staphylococcus aureus*, and *Escherichia coli*. The results showed that *KP* exhibited a distinct fluorescent signal, while other strains showed almost no background fluorescence, confirming the specificity of the system (**Figure 5B**).

### 3.6 The detection of clinical strains by light-controlled one-pot RPA-CRISPR/Cas12a method

**Figure 6:**
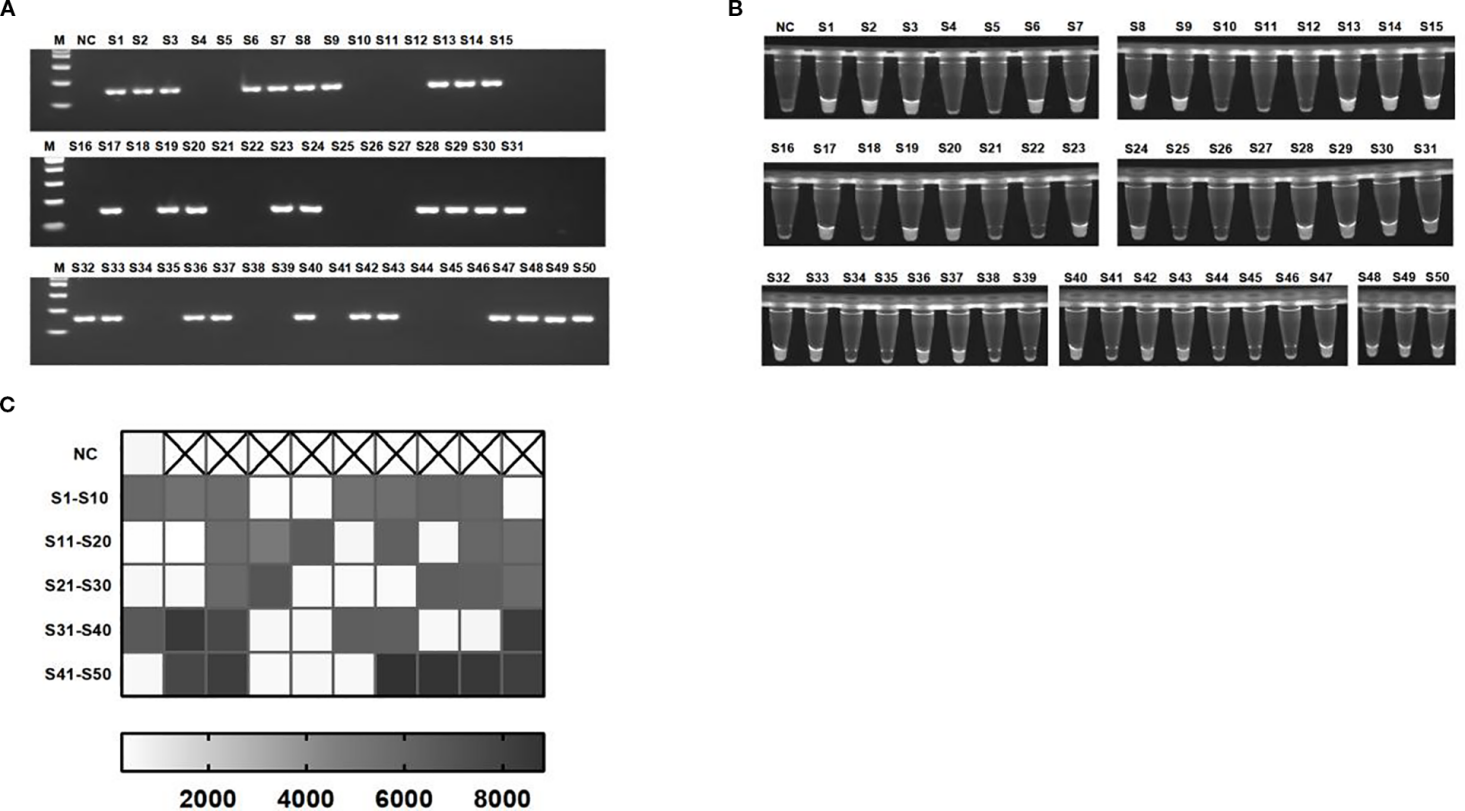
Detection of *KP* clinical strains. **(A)** Representative 2% agarose gel electrophoresis confirming PCR-based identification of clinical isolates. **(B, C)** Visualization signals and endpoint fluorescence value heatmap of 50 clinical strains detected using the light-controlled one-pot RPA-CRISPR/Cas12a method. NC, negative control. S1-S50, all strains are derived from clinical sources.

In this study, 30 clinical strains of *KP* and 20 negative control strains were used as samples to compare and analyze the detection performance of the light-controlled RPA-CRISPR/Cas12a integrated detection system and the traditional PCR method, and the sensitivity and specificity of the technology in clinical application were systematically evaluated. The results of PCR analysis showed that among the 50 clinically isolated strains, 30 strains successfully amplified the *rcsA* gene fragment, and were identified and confirmed to be *KP* (positive detection rate of 60%). The remaining 20 strains were judged to be control colonies because the *rcsA* gene was not detected (**Figure 6A**). The light-controlled one-tube RPA-CRISPR/Cas12a detection system was further verified, and the ultraviolet signal analysis showed the detection results were in complete agreement with the PCR method (100% coincidence rate) (**Figures 6B, C**). The receiver operating characteristic (ROC) curve shows that at a fluorescence threshold of 2710, the area under the ROC curve (AUC) is 1.000, with sensitivity and specificity of 100% (95% confidence interval: 88.65–100%) and 100% (95% confidence interval: 83.89–100%), respectively (**Supplementary Figure S1** **).**

## 4 Discussion

*KP* is the main causative agent of pneumonia, urinary tract infections, and bacteremia, and is an important opportunistic healthcare-associated pathogen. In 2017, the World Health Organization recognized the prevalence of its multidrug-resistant strains as a serious public health threat (Stojowska-Swędrzyńska et al., 2021). This strain is not only an important vector for the global spread of antibiotic resistance, but also a major risk factor for infection due to colonization of the gastrointestinal tract, as well as a key hub for the spread of drug resistance (Lindstedt et al., 2022). Due to the high multidrug resistance of *KP* strains, which complicates infection treatment (Kim et al., 2022), there is an urgent need for rapid and accurate diagnostic methods to enable early prevention. This would effectively curb the spread of drug-resistant bacteria and improve clinical outcomes.

Although the traditional bacterial culture method is regarded as the gold standard for the diagnosis of *KP*, its diagnosis cycle is long, usually 48 to 72 hours, which is extremely detrimental to the timely treatment and disease control of patients. Although the detection time of the PCR method is shorter than that of traditional bacterial culture methods, it has higher requirements for instruments and equipment and needs to be performed by professionally trained operators, which limits its wide application to a certain extent. RPA is an isothermal nucleic acid amplification technology that has attracted much attention in recent years. This technology is characterized by easy operation, fast amplification speed (usually completed in 20–30 min), and mild reaction temperature (37-42 °C) (Tan et al., 2022; Liao et al., 2024). As a result, rapid amplification of the gene of interest can be achieved. The CRISPR/Cas12a system has the advantages of high specificity and high sensitivity. Therefore, the integration of RPA and CRISPR systems can achieve rapid and efficient detection of target genes (Aman et al., 2020, 2021; Zhang et al., 2023; Ji et al., 2025). This study successfully constructed a “one-pot” RPA detection platform based on photo-controlled CRISPR/Cas12a. By introducing NPOM modified crRNA, rapid detection of *KP* was achieved (detection time of 50 minutes, sensitivity of 4.072 × 10^2^ copies/reaction). As a photocleavable group, NPOM-dt modification temporarily inactivates crRNA (caged crRNA), inhibiting Cas12a activity. Upon UV irradiation of caged crRNA, NPOM-dt dissociates, restoring crRNA activity to guide Cas12a in recognizing target DNA and triggering its cleavage function. The entire detection process enables a “one-pot” operation without requiring additional reagents. This feature is particularly advantageous for developing freeze-dried reagents, significantly enhancing the portability of detection kits.

Compared to the traditional two-step RPA-CRISPR/Cas12a detection system, this method employs a single-tube reaction format, significantly reducing the risk of aerosol contamination associated with the transfer of amplified products (Tan et al., 2024b; Wu et al., 2025). Experimental results demonstrate that the light-controlled “one-pot” assay exhibits comparable performance to the standard two-step method, achieving identical sensitivity (10–^6^ ng/μL) and specificity (100%) (Zhou et al., 2024; Wei et al., 2025). These findings indicate that the performance of the developed detection system is unaffected by single-tube operation. In the traditional one-step method, the CRISPR system activates and cleaves the template early in the RPA amplification, which affects the accumulation of target amplification products, thus reducing the sensitivity of the detection system (Gao et al., 2024; Zhang et al., 2024). This study successfully avoided premature activation of the CRISPR system by employing a light-controlled temporal regulation mechanism, thereby achieving high efficiency and reliability in single-tube reactions.

Compared to recent studies, this detection system demonstrates significant advantages in operational convenience, cost-effectiveness, and detection efficiency. The method developed by Fu et al. enables detection within 31 minutes (with a detection limit as low as 10^1^ CFU/μL) (Fu et al., 2025). However, it requires screening multiple suboptimal PAM crRNAs, increasing detection costs. In contrast, our system requires only a 15 μL reaction volume (reducing standard RPA volume by four-fifths) and utilizes standard UV lamp equipment costing just $15, making the overall cost more competitive. Recently, Wu et al. reported that they have achieved rapid detection of *KP* (40 minutes, detection limit 10^0^ copies/μL) through a extraction-free, tube-cap, “one-pot” RPA-CRISPR/Cas12a method (Wu et al., 2025). The simplicity and rapidity of the assay are augmented by a straightforward sample processing without extraction. In contrast, this study does not require separate regulation of RPA and CRISPR steps, but DNA extraction increases the time cost and operational complexity. Furthermore, Wang et al.’s PCR-CRISPR/Cas12a detection method takes approximately 2 hours (Wang et al., 2023); Zhang et al.’s LAMP-CRISPR/Cas12b system (Zhang et al., 2025) requires constant 58 °C incubation and multiple primer sets, further increasing instrumentation requirements and reagent costs.

During experimental optimization, we successfully enhanced detection performance through systematic adjustments to RPA primer concentration, illumination duration, Cas12a protein concentration, and caged crRNA concentration. The experiment of missing reaction components has demonstrated the feasibility of a one-pot RPA CRISPR/Cas12a detection system controlled by light, which only triggers the reaction under illumination. Following the method described by Hu et al. (Hu M. et al., 2023), we employed a 35 W, 365 nm UV lamp for 30 seconds of irradiation to ensure complete shedding of NPOM-dt. Given the brief exposure duration and high DNA concentration post-isothermal amplification, any potential DNA damage from UV exposure is negligible within the detection system.

Nevertheless, this study has the following limitations and directions for future improvement: Firstly, clinical applicability of UV irradiation: Although a commercial LED UV lamp with a fixed wavelength (365 nm) was used, activation still relies on manual timing control. Future development will focus on integrating a portable UV-regulated module into the fluorescent detection device to enhance operational standardization and clinical applicability. Secondly, sample size limitation: Although results from 50 clinical samples showed complete concordance with PCR, the small sample size still indicated that this study was a proof-of-concept and needs to be validated in a larger sample size before being put into clinical application in the future. Thirdly, DNA extraction dependency: Sample pretreatment is required, limiting point-of-care testing applications. Although DNA extraction-free methods exist (Wu et al., 2025), they involve complex procedures and higher costs. Future development will focus on developing a simple, low-cost, light-controlled, extraction-free, single-pot detection system to advance point-of-care testing applications.

In conclusion, our study proposes a one-tube detection system for the detection of *KP* using light-controlled CRISPR/CAS12a combined with RPA, which has significant advantages in preventing aerosol contamination and speed, sensitivity, and portability. This platform facilitates the rapid detection of the *rcsA* gene in *KP*. Its applicability in the early and rapid diagnosis of *KP* infection is expected to curb its potential for widespread spread, particularly in hospital settings. In addition, the system provides a cost-effective screening method tailored to resource-constrained areas.

## Data Availability

The datasets presented in this study can be found in online repositories. The names of the repository/repositories and accession number(s) can be found in the article/**Supplementary Material**.
