# Supplementary material for: Klebsiella pneumoniae detection by a light-controlled one-pot RPA-CRISPR/Cas12a method
Source: Front Cell Infect Microbiol. 2025 Oct 1;15:1669860. doi: 10.3389/fcimb.2025.1669860 (PMC12521164; doi:10.3389/fcimb.2025.1669860)
Supplement: Supplementary file 1 [file DataSheet1.pdf]

Table S1. Information on pathogenic microorganisms used in this study

Name	Category number	Source
K.pneumoniae	ATCC700603	American Type Culture Collection
S.aureus	ATCC29213	American Type Culture Collection
E.faecalis	ATCC29212	American Type Culture Collection
E.coli	ATCC25922	American Type Culture Collection
S. pneumoniae	ATCC49619	American Type Culture Collection
P.aeruginosa	ATCC27853	American Type Culture Collection
E.faecium	GDMCC1.388	Guangdong Microbial Culture Collection Center
H. parainfluenzae	-	Clinical isolates

Table S2. Information on the equipment used in this study

Equipment name	Source
Tianlong Gentier 96E/96R qPCR instrument	Xi'an, China
Amersham Imager 600	General Electric Healthcare, USA

Table S3. Sequences of Primers, crRNA, and ssDNA reporters involved in this study

Primer	base sequence	base number
KP-rcsA Pair1	F:CGCTGGCGAACATCCATTTTGACCGCTATTTG<br>R:CTGAATACCGGAGGTGATGTTTTCGGTCAGCC	32
KP-rcsA Pair2	F:ACCCGGCGACGCTGTTTGTTATCTTTATGT<br>R:GATACCGTCTTCGCTTTGATGTTCATTTGC	30
KP-rcsA Pair3	F:TAACCCCAAAAGACCTTGATGTTATTC<br>R:TCACCTGCTTATTATGCGTTTGTATTT	27
KP-rcsA Pair4	F:TGGATTTGTGCAGCTATACCCGGTTGGGATTG<br>R:CGCAAATAGCGGTCAAAATGGATGTTCGCCAG	32
crRNA1	UAAUUUCUACUAAGUGUAGAUCUCAGUGACAAUGUCGGUAA	41
crRNA2	UAAUUUCUACUAAGUGUAGAUAUGUUCAUUUGCGUUGAGAU	41
crRNA3	UAAUUUCUACUAAGUGUAGAUCGAAGUACCAUGCCCGGCCA	41
PCR-primer	F:GGCATGGTACTTCGCAAATCTC<br>R:CCTGAATACCGGAGGTGATGTT	22
SSDNA	5‘FAM-TTATT-BHQ1’3<br>5‘FAM-TTATT-biotin’3	6
Caged crRNA	UAAUUUCUACUAAGUGUAGAUCUCAGUGACAAUGUCGGUAA	41

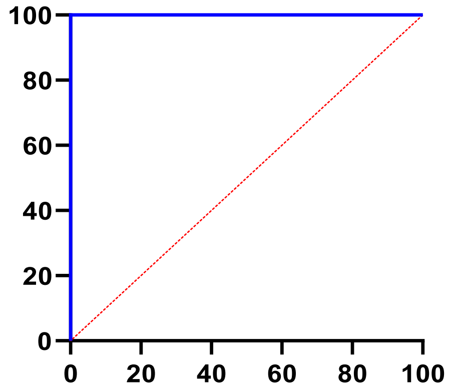

Figure S1. Evaluate the performance of RPA-CRISPR/Cas12a detection using ROC curve.
